#  Relationship between MRI quantified small bowel motility and abdominal symptoms in Crohn’s disease patients—a validation study

**DOI:** 10.1259/bjr.20170914

**Published:** 2018-06-18

**Authors:** Ruaridh M Gollifer, Alex Menys, Jesica Makanyanga, Carl AJ Puylaert, Frans M Vos, Jaap Stoker, David Atkinson, Stuart Andrew Taylor

**Affiliations:** 1 Centre for Medical Imaging, University College London (UCL), London, United Kingdom; 2 Department of Radiology and Nuclear Medicine, Academic Medical Center (AMC), Amsterdam, Netherlands; 3 Quantitative Imaging Group, Delft University of Technology, Delft, Netherlands

## Abstract

**Objective:**

Previous single-centre MRI data suggests an inverse correlation between normal small bowel motility variance and abdominal symptoms in Crohn’s disease (CD) patients. The current work prospectively assesses this observation in a larger, two-centre study.

**Methods:**

MR enterography datasets were analysed from 82 patients (38 male, aged 16–68), who completed a contemporaneous Harvey-Bradshaw index (HBI) questionnaire. Dynamic “cine motility” breath-hold balanced steady-state free precession sequences were acquired through the whole small bowel (SB) volume. Regions of interest (ROIs) were manually applied to encompass all morphologically normal SB (*i.e.* excluding Crohn’s affected bowel) and a validated registration technique used to produce motility maps. Mean and variance motility metrics were correlated with HBI and symptom components (well-being, pain and diarrhoea) using Spearman’s correlation statistics.

**Results:**

Overall, motility variance was non-significantly negatively correlated with the total HBI score, (*r* = −0.17, *p* = 0.12), but for subjects with a HBI score over 10, the negative correlation was significant (*r* = −0.633, *p* = 0.027). Motility variance was negatively correlated with diarrhoea (*r* = −0.29, *p* < 0.01). No significant correlation was found between mean motility and HBI (*r* = −0.02, *p* = 0.84).

**Conclusion:**

An inverse association between morphologically normal small bowel motility variance and patient symptoms has been prospectively confirmed in patients with HBI scores above 10. This association is particularly apparent for the symptom of diarrhoea.

**Advances in knowledge:**

This study builds on preliminary work by confirming in a large, well-controlled prospective multicentre study a relationship between normal bowel motility variance and patient reported symptoms which may have implications for drug development and clinical management.

## Introduction

Crohn’s disease (CD) is a lifelong chronic inflammatory bowel disease characterised by periods of disease activity and remission.^1^ Current treatments, typified by antiTNF alpha agents, are directed at effectively managing inflammatory activity with the aim of both treating patients’ symptoms and reducing long-term bowel damage.^2^

Patient symptoms remain an important outcome measure for judging disease control. The Harvey-Bradshaw index (HBI) for example is a long established and validated questionnaire designed to quantify patient symptoms, which can be readily employed in routine clinical practice. The HBI consists of five components including general well-being, abdominal pain, number of liquid stools per day, abdominal mass and complications and patients’ disease activity is classified based on their total score.^3^

It is however clear that symptom burden is not always directly related to inflammation.^4, 5^ For example faecal calprotectin levels and MRI features of bowel inflammation often show poor correlation with the HBI.^6^ Furthermore, even when patients are in apparent endoscopic and biochemical remission, many still suffer ongoing abdominal symptoms including diarrhoea and abdominal pain with profound effects on quality of life.^4, 7,8^

The aetiology of abdominal symptoms and their link to disease activity therefore remains obscure. Recently, quantification of segmental and global small bowel motility using MRI is providing novel insights into CD pathophysiology.^9–14^ Indeed, recent single-site data in 53 patients suggests that aberrant motility (notably a decreased variability) in apparently healthy bowel may be linked to patient symptom burden in CD.^15^ If this observation could be prospectively validated, MRI quantified bowel motility could provide new insights into the aetiology of abdominal symptoms in CD, act as a target for therapeutic monitoring and possibly aid drug development.

The purpose of this study was to validate the apparent link between reduced small bowel motility variance and patient abdominal symptoms in CD as part of a prospective multicentre study.

## Methods and Materials

### Patient selection

The current study was approved by both centres’ ethics committees (**Hampstead REC, London and the ethics committee of the Academic Medical Center**), and all patients gave written informed consent, and details a subset of patients recruited to the VIGOR++ study, a prospective trial which developed novel software metrics to quantify CD activity using MRI in comparison to an endoscopic reference.^16^ In brief, patients with known or suspected CD underwent contemporaneous (within 2 weeks) MR enterography and colonoscopy at two centres (Academic Medical Centre, AMC and University College London Hospital, UCLH) between October 2011 and September 2014. As part of the trials, patients completed a HBI symptom questionnaire the day prior to scanning^3^ (Supplementary material 1). Demographic data pertaining to age, sex, current medication, disease duration and surgical history was also collected.

Patients were excluded from the current study if they had either failed to undergo an adequate dynamic MRI cine sequence through the whole small bowel volume (greater than three slices and complete time series - see MRI protocol details below), failed to complete a HBI questionnaire or were taking medication with known direct effects on motility such as prokinetic agents (for example neostigmine) and anti-spasmodics (for example buscopan). Exclusions were confirmed a priori and before data analysis.

### MRI protocol

Patients fasted for 4 h before ingesting 800 ml of 2.5% mannitol, 3 h prior to the start of the scan to distend the colon. A further 1600 ml of 2.5% mannitol was provided 1 h before the scan start time to distend the small bowel.

Patients were scanned in the supine position on 3T systems (Ingenia/Achieva; Philips, Best, Netherlands) using the manufacturer’s torso array coil. In addition to standard anatomical sequences (*T*_2_ single-shot fast spin echo and *T*_1_ spoiled gradient echo) a dynamic “cine motility” sequence was acquired during a 22 s breath-hold prior to spasmolytic administration using a two-dimensional (2D), coronal, balanced steady-state free precession sequence with the following parameters: flip angle 45^o^, repetition time = 2 ms, echo time = 1 ms, 256 × 200 matrix filling, zero-filling to 512 × 512 and 1 × 1 in plane resolution, temporal resolution = 1.1 s, slice thickness = 10 mm. The MRI radiographer/technician repeated these coronal blocks to encompass the whole small bowel volume, the number of acquisitions ranging from 5 to 14 depending on the size of the patient.

### Motility assessment

#### Step 1: Generation of the SD Jacobian

**Figure 1.**
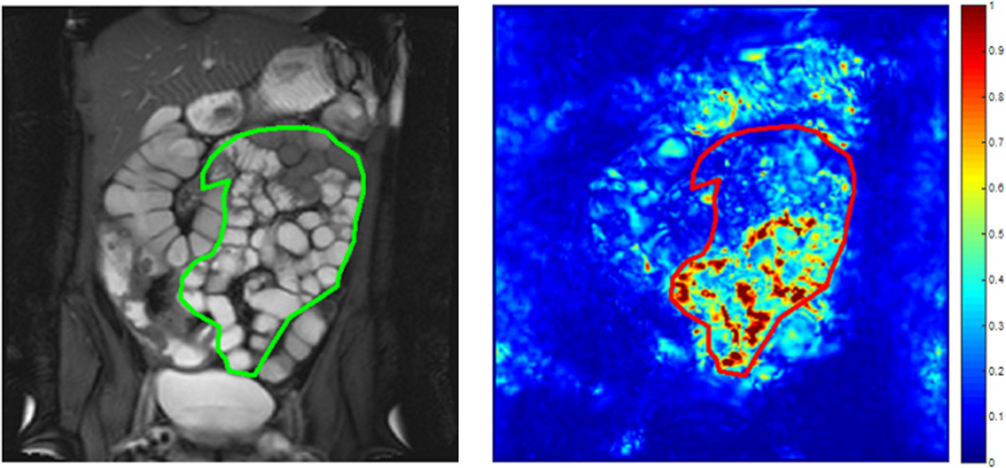
An example of a coronal reference frame displaying a SB ROI (left) with the corresponding motility map of the whole small bowel volume (right), based on the SD of the Jacobian determinant. This example demonstrates heterogeneous motility with areas of active (red) and inactive (dark blue) SB. ROI, region of interest; SB, small bowel; SD, standard deviation.

Frames from the 2D cine motility sequence were registered using a previously validated optic-flow-based registration technique.^17^ The standard deviation (SD) of the Jacobian determinant of the deformation fields was calculated and will henceforth be referred to as the SD Jacobian.^17^ This summarises the variations in time of local expansion and contraction on a per pixel basis and is displayed on a reference frame (or motility map), automatically selected from the time series images by the registration algorithm (Figure 1). The motility map therefore essentially displays the SD Jacobian over time, which represents the area change of each pixel in the image. The movement of pixels within manually placed regions of interest (ROIs) is quantified by the registration software as a surrogate for bowel motility.^15, 17,18^

For the current study, a graphical user interface (MATLAB, MathWorks, Natick, MA) was used for motility analysis. Within the viewer, anonymised datasets are displayed, as both a static reference image and as a “cine” movie.

#### Step 2: Motility analysis and ROI placement

For each patient, a research fellow with 6 months training in enteric MRI (**RMG**), used the viewer to place ROIs on the reference image (without motility map displayed), blinded to the HBI score. The ROIs were validated by a research fellow with over 5 years MRE experience (**AM**).

The ROIs were deliberately placed in morphological normal small bowel only, excluding small bowel affected by CD.^15^ The observers had access to both cine motility loops and anatomical small bowel images to aid ROI placement. Specifically, small bowel demonstrating the typical stigmata of CD (such as wall thickening, abnormal *T*_2_ signal hyper enhancement etc.)^19^ was excluded from the ROI, as was the small bowel mesentery. ROIs were placed in each of the individual cine motility blocks acquired for each patient so as to include all morphologically normal bowel as far as possible.

Based on the previous derivation study,^15^ two motility metrics were derived from the ROIs and summed across the whole patient: (1) mean and (2) spatial variance. The original motility measure derived from registration *i.e.* SD Jacobian provides a measure of expansion and contraction over time at each pixel.^17^ The two motility metrics here provide summaries over the ROIs of their spatial distribution.

**Figure 2.**
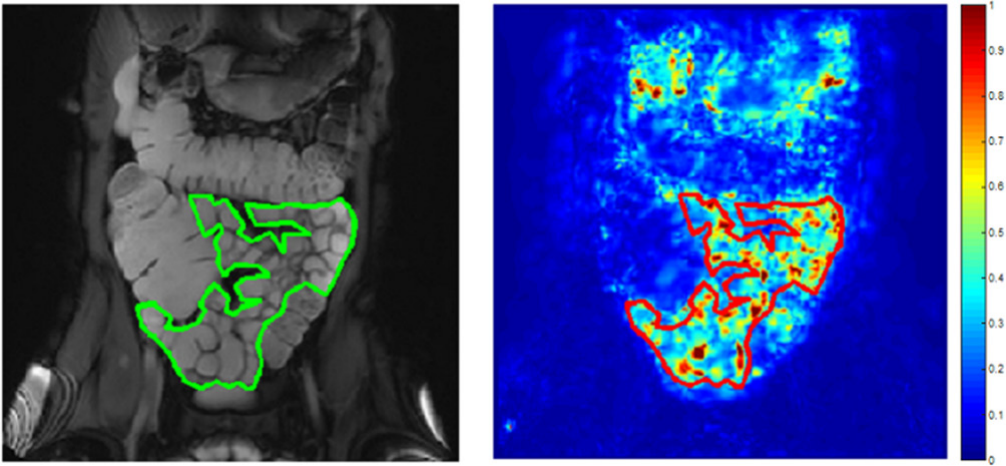
An example of high homogeneous motility where the small bowel in the ROI is predominantly motile. ROI, region of interest.

The *mean* motility metric *i.e.* mean SD Jacobian gives an indication of the overall motility of the segmented bowel with a high value suggesting high motility (Figure 2).

**Figure 3.**
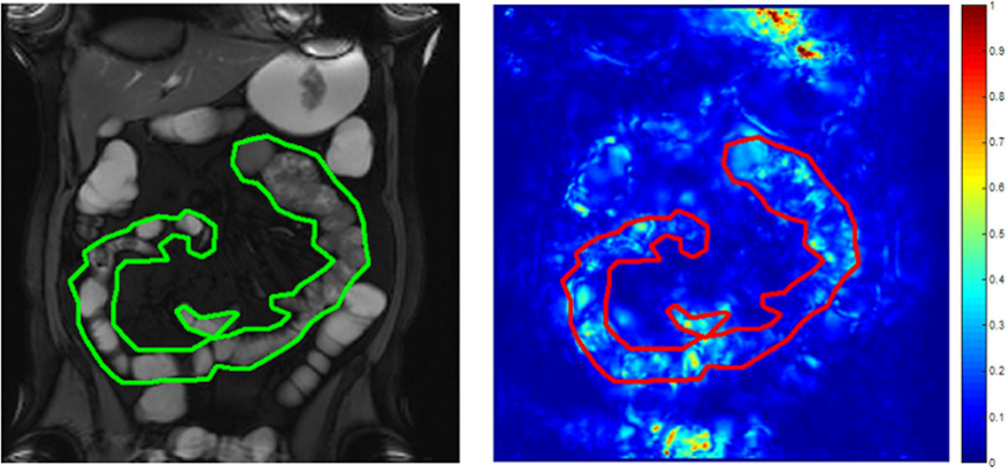
An example of low homogeneous motility where the small bowel in the ROI is predominantly immotile. ROI, region of interest.

The motility *variance* metric *i.e.* variance of SD Jacobian gives an indication of the spatial variability of motility *e.g.* high motility variance corresponds to a wide range of SD Jacobian values across the small bowel with areas of both high and low motility, independent of the overall motility level (Figure 1). Conversely a low motility variance corresponds to more homogeneous motility with less variation in the range of SD Jacobian values. For example, a patient with low motility variance could have either homogenously high (Figure 2) or homogeneously low motility (Figure 3).

### Statistical analysis

All statistical analysis was performed using MATLAB (MathWorks).

All data were checked for normality using Shapiro-Wilk test (alpha = 0.05); non-parametric statistics were used in cases where data were non-normally distributed.

Correlation was performed between the two motility metrics and the total HBI symptoms score using Spearman’s correlation statistics, with *p* ≤ 0.05 being taken as statistically significant.

The best performing metric was then correlated with each of the HBI subcomponents of well-being, pain and liquid stools, again using correlative statistics.

An additional analysis was restricted to those with a HBI above certain thresholds. Two thresholds were tested; a HBI score of 8 or higher and 10 or higher. The lower threshold (HBI ≥8) was selected as a score of 8–16 is defined as moderate CD activity, with severe disease assumed with a HBI above 16^3^. The higher threshold (HBI ≥10) was chosen as the maximum score for the subjective measures of general well-being, abdominal pain and abdominal mass is 10. Therefore, for a patient to achieve a score of 10 or above they will likely need to score highly on objective measures such as liquid stools.

## Results

### Cohort demographics

The full VIGOR++ study cohort consisted of 158 patients (89 AMC, 69 UCLH).

For the current study, a total of 76 patients were excluded for the following reasons; diagnosis other than CD (*n* = 18), >14 days between MRI and colonoscopy (*n* = 7), failure to comply with oral contrast protocol (*n* = 6), cancelled or aborted ileocolonoscopy (*n* = 5), missing motility sequences or inadequate small bowel coverage (*n* = 14), acquired motility sequences data was not available for analysis (*n* = 24), insufficient bowel cleansing (*n* = 1) and non-compliance to breathing commands due to a language barrier (*n* = 1).

The demographics for the remaining 82 patients (41 BLIND, 41 BLIND) included in the current study are shown in Table 1.

**Table 1.** Patient demographics with 82 patients in study cohort

**Parameter**	**AMC**		**UCLH**	
Age	19–68 years old (median age 35)		16–63 years old (median age 29)	
Males (%)	22 (54%)		16 (39%)	
Disease duration (years)	<1 1–5 5–10 >10 Unknown	4 4 11 21 1	<1 1–5 5–10 >10 Unknown	4 8 16 12 1
Disease location	Ileal Colonic Ileocolonic	22 6 13	Ileal Colonic Ileocolonic	6 7 28
Medications	None 5-ASA Immuno- modulators Biological Agents	7 3 15 16	None 5-ASA Immuno- modulators Biological Agents	10 18 14 9
Surgical history	None 1 operation 2 operations	22 13 6	None 1 operation 2 operations	31 10 0

### HBI and motility metrics

The range and median values of the two motility metrics and HBI score is shown in Table 2. Median total HBI score for the cohort was 5, ranging from 0 to 38. For patients recruited from UCLH, the median was 4, ranging from 0 to 10 and for patients recruited from AMC, the median was 7, ranging from 0 to 38.

**Table 2.** Median, minimum and maximum motility and HBI values

	Median	Range	
Minimum	Maximum
**Mean motility metric**	0.34	0.16	0.51
**Motility variance metric**	0.038	0.012	0.085
**HBI**	5	0	38
* Well-being*	1	0	4
* Abdominal pain*	1	0	3
* Liquid stool*	2	0	30

HBI, Harvey-Bradshaw index.

**Figure 4.**
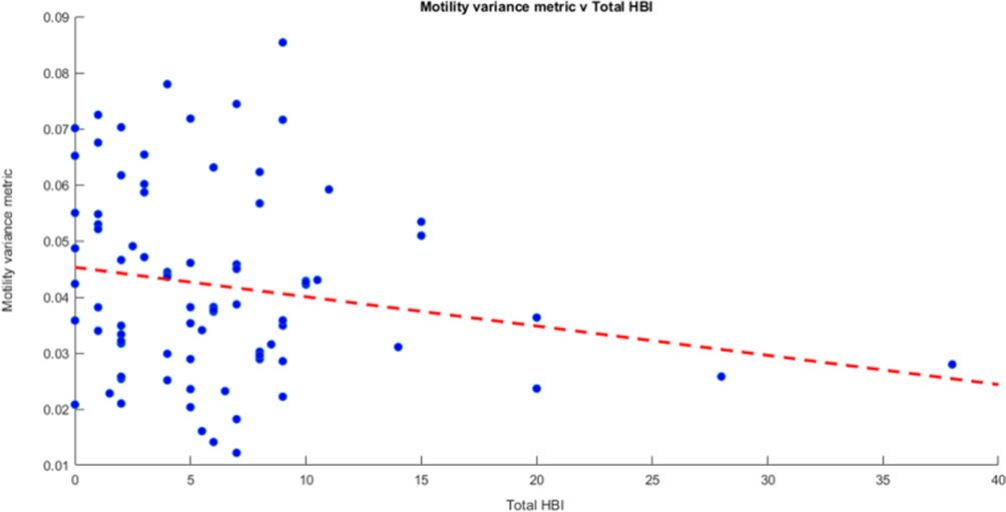
Motility variance metric *vs* total HBI. There was some evidence of an inverse association between motility variance metric and the total HBI (*r* = −0.17, *p* = 0.12). HBI, Harvey-Bradshaw index.

There was a negative correlation between the motility variance metric and total HBI score, although this did not reach statistical significance (*r* = −0.17, *p* = 0.12) (Figure 4). There was no evidence of any correlation between the mean motility metric and total HBI score (*r* = −0.02, *p* = 0.84). The motility variance metric was therefore the best performing metric.

### Motility variance metric against total HBI with threshold

**Figure 5.**
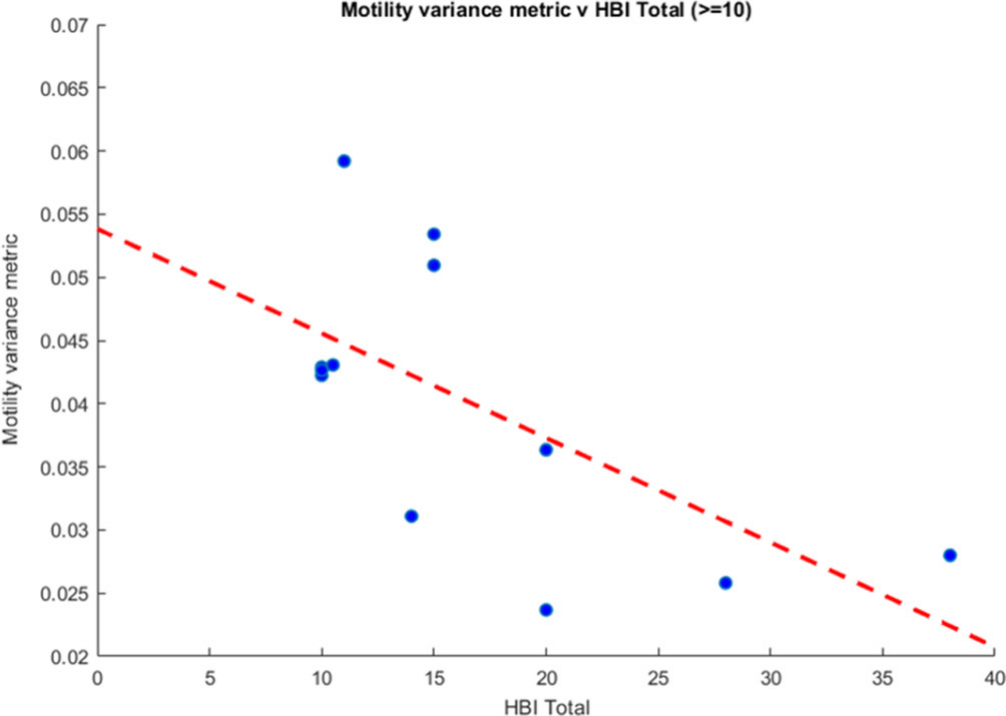
The motility variance metric *vs* HBI scores of 10 or above. There was a significant correlation (*r* = −0.633, *p* = 0.027). HBI, Harvey-Bradshaw index.

When a threshold of 10 or above was applied to the HBI score (*i.e.* high patient symptom load), there was a significant negative correlation between the motility variance metric and HBI (*r* = −0.633, *p* = 0.027) (Figure 5). There was a negative correlation between the motility variance metric and a HBI score of 8 or above, but this was weaker and not significant (*r* = −0.276, *p* = 0.18).

### Motility variance metric against HBI components

**Figure 6.**
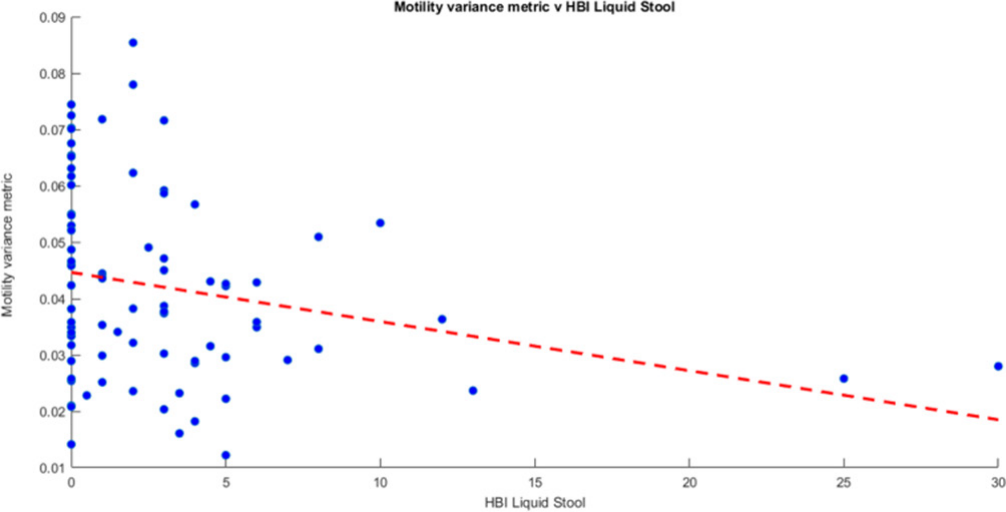
Motility variance metric *vs* HBI liquid stools. There was a significant inverse association between the motility variance metric and the HBI liquid stools (*r* = −0.29, *p* < 0.01). HBI, Harvey-Bradshaw index.

There was also a significant negative correlation between the motility variance metric and the number of diarrhoeal stools (*r* = −0.29, *p* < 0.01) (Figure 6). Conversely there was no correlation between the motility variance metric and the other HBI components of pain and well-being (Table 3).

**Table 3.** Correlation of HBI components (diarrhoeal stools, pain and well-being) against motility variance metric

HBI component	*r* value (correlation against motility variance metric)	*P* value (correlation against motility variance metric)
**Diarrhoeal stools**	−0.29	<0.01
**Pain**	−0.022	0.85
**Well-being**	0.023	0.84

HBI, Harvey-Bradshaw index.

## Discussion

Our prospective data demonstrates an inverse association between motility variance in morphologically normal small bowel and high patient symptom burden in CD. No correlation was found with mean motility, indicating that absolute levels of motility are not a significant driver of patient reported symptoms.

Previously, using a single-centre study design in 53 Crohn’s subjects, Menys et al^15^ reported a significant inverse correlation between motility variance and HBI (*r *= –0.45, *p* < 0.001). There was also a significant inverse correlation between motility variance and the HBI symptom components of well-being, abdominal pain and number of liquid stools (*r* = −0.4, *p* < 0.01).

This current prospective validation study in part reproduces these findings, although the associations were weaker. We again found a significant correlation between motility variance and total HBI, but only in patients with a HBI score of 10 or above. We also again found an inverse association between motility variance and the symptom of diarrhoea but no correlation with symptoms of well-being or abdominal pain. Indeed it seems that the symptom of diarrhoea is the major contributor to the observed inverse correlation between motility variance and HBI (we did not find associations with the individual scores of pain and well-being).

The reason for this apparent inverse association between motility variance and patient symptoms is unclear and prospective mechanistic studies are underway. A possible explanation is that impaired coordination of bowel motility rather than changes in absolute levels leads to worsening of patient symptoms, particularly diarrhoea. Variation in small bowel motility is a normal finding in healthy individuals^20–24^ and appears to be a marker of gut well-being. A reduction in this variability in turn may lead to abdominal symptoms. The post-prandial state involves peristalsis and segmentation to facilitate the mixing of food ingested and absorption of nutrients. In this state however, variability is induced across the bowel volume, with episodes of peristalsis movement interspersed with periods of inactivity which prolongs transit time to aid absorption.^20, 25,26^ The mannitol administered prior to MR enterography seems to mimic the post-prandial state,^27^ allowing us to capture the complexities of gut motility in a controlled and reproducible way.^18^ We can surmise that because mean motility scores were not correlated to diarrhoeal stools, that absolute bowel motility is unlikely to be the driver for symptoms.

It is interesting to note that validated MRI CD activity scores which are based on structural observations such as bowel wall thickening, T_2_ signal and contrast enhancement etc. in the main show no association with clinical indices to assess symptoms such as HBI. For example, the CD activity score has been developed and validated against a histological standard of reference^28^ and has recently been extended to provide a global MRI activity score (MRI enterography global score).^6^ However, in previous work no significant correlation was found between the MRI enterography global score and HBI (*r* = 0.102, *p* = 0.40) in a cohort of 71 patients.^6^ Additionally, Makanyanga et al^6^ reported no correlation between a CD activity score and HBI (*r* = 0.045, *p* = 0.630). Indeed, HBI also correlates poorly with objective measures of inflammation such as faecal calprotectin (fCP). Sipponen et al^29^ for example showed no correlation between HBI and fCP (*p* > 0.05).

This suggests the HBI score reflects more than simply underlying CD inflammatory activity and there are alternative drivers behind patient symptoms, potentially including aberrant motility.^6, 30^ It also suggests that anatomical or structural MRI observations show little correlation with patient symptoms as captured by HBI. It has been acknowledged however that we did not reconfirm this lack of association as part of the design of the current study.

It should also be acknowledged that the HBI has limitations as a method to capture patient symptoms. More complex CD questionnaires such as the Inflammatory Bowel Disease Questionnaire have been developed.^31^ However, the three symptoms encompassed by the HBI are clearly of great importance to patients. Of course, whatever tool is used to capture symptoms, they are by their very nature subjective. In the context of HBI, two patients experiencing a similar level of pain could class this symptom as mild to severe depending on their individual perception.

We found that the association between reduced spatial motility variation and patient symptoms was strongest at the higher end of HBI scores *i.e.* in those with a greater symptom burden.

To attract a high HBI score of 10 or above, patients usually need to record a high level of diarrhoeal stools, which is arguably a more objective measure of patient symptomatology compared to the more subjective pain or well-being scores. Conversely, the correlation between HBI and reduced motility variance was poor for lower HBI scores, with a large range of motility variance, and poor separation between patients in remission (<5 HBI) and those with mild/moderate CD “activity” (5–10 HBI). It would perhaps be expected that CD patients with mild symptoms would be much more likely to exhibit heterogeneous motility (with high motility variance) since they are presumably closer to having “healthy bowel”.

The utility of motility metrics in future mechanistic research may therefore be greatest in patients with moderate and severe abdominal symptoms. Future research will investigate the effects of Crohn’s medication on motility indices and patient symptoms. The group of patients who retain a high symptom burden despite apparently being in clinical remission are of particular interest, as it may be that aberrant motility, if present, may be a target for pharmacological intervention.

Our study does have limitations. The HBI threshold of 10 was chosen for subanalysis as this value, by definition, includes a contribution from objective measures such as liquid stools due to the nature of the HBI scoring system. We acknowledge the more traditional cut off for moderate disease is 8 and for severe is over 16^3^.

We interrogated only morphologically normal bowel on MRI criteria. We acknowledge that in the absence of capsule endoscopy or histological sampling, we cannot be absolutely certain that subtle CD was not present.

We only acquired motility data over a 20 s breath-hold which may not be sufficient to capture the true complex nature of bowel motility. In future, it might be more desirable to acquire longer free breathing datasets. This would reduce patient discomfort compared to breath-holding and potentially capture a more complete picture of motility. Software has already been developed which can correct for respiratory motion.^32^ The MRI motility protocol involved acquiring multiple 2D slices, each consisting of a time series, to obtain full coverage of the bowel. However, these were acquired at different times so there was a temporal incoherence between slices which were acquired 30 s apart.

It should be noted that a reasonable proportion of the original 158 datasets were excluded (*n* = 76 excluded). However, only 14 of these were due to an incomplete MRI protocol (*e.g.* missing sequences or inadequate small bowel coverage) and the rest was due to HBI or MRI data not being available. Therefore, only a small proportion of the excluded datasets were caused by difficulty of acquiring dynamic MRI or poor image quality.

## Conclusion

In summary, this prospective study has demonstrated an inverse relationship between normal small bowel motility variance and patient abdominal symptoms in CD, particularly diarrhoeal stools. The association is strongest in patients with HBI scores greater than 10.
